# Development of a Prototype Over-Actuated Biomimetic Prosthetic Hand

**DOI:** 10.1371/journal.pone.0118817

**Published:** 2015-03-19

**Authors:** Matthew R. Williams, Wayne Walter

**Affiliations:** 1 Louis Stokes Cleveland Department of Veterans Affairs Medical Center, Cleveland Functional Electrical Stimulation Center, Cleveland, Ohio, United States of America; 2 Department of Mechanical Engineering, Rochester Institute of Technology, Rochester, New York, United States of America; University of Chicago, UNITED STATES

## Abstract

The loss of a hand can greatly affect quality of life. A prosthetic device that can mimic normal hand function is very important to physical and mental recuperation after hand amputation, but the currently available prosthetics do not fully meet the needs of the amputee community. Most prosthetic hands are not dexterous enough to grasp a variety of shaped objects, and those that are tend to be heavy, leading to discomfort while wearing the device. In order to attempt to better simulate human hand function, a dexterous hand was developed that uses an over-actuated mechanism to form grasp shape using intrinsic joint mounted motors in addition to a finger tendon to produce large flexion force for a tight grip. This novel actuation method allows the hand to use small actuators for grip shape formation, and the tendon to produce high grip strength. The hand was capable of producing fingertip flexion force suitable for most activities of daily living. In addition, it was able to produce a range of grasp shapes with natural, independent finger motion, and appearance similar to that of a human hand. The hand also had a mass distribution more similar to a natural forearm and hand compared to contemporary prosthetics due to the more proximal location of the heavier components of the system. This paper describes the design of the hand and controller, as well as the test results.

## Introduction

Current estimates of upper extremity place the number of individuals with major limb loss (extremity loss more significant than fingers or toes) in the U.S. at 1.7M people [1]. Of these, approximately 41,000 are upper limb amputees. While 80% of all amputations are the result of vascular disease, upper extremity amputations are primarily (69%) the result of trauma. Despite upper extremity loss resulting in substantially more disability than lower extremity amputation [2], this relatively small pool of potential users, and the ensuing lack of return on investment, may be one reason why upper extremity prosthetics have changed relatively little since the 1950’s [3,4]. That said, recent conflicts in Iraq and Afghanistan have swelled the number of amputees, with approximately 22% of those who have lost a limb in combat having at least one upper extremity amputation more significant than loss of fingers [4–6]. As was the case with previous wars and conflicts, this increased occurrence of combat-related upper extremity amputation has raised overall interest in the development of more advanced prosthetic hands [7,8]. As more veterans return home with upper limb amputations, the need for more capable hand prostheses has become critical to helping them to regain their former lives. There exists a very real need for prosthetics that are more adept and flexible to more fully meet user needs better than currently available clinical options. The purpose of this work is to present a prototype of a novel, biomimetic upper extremity prosthetic hand design that addresses some of the issues raised by users while remaining an acceptable device.

Currently available prosthetic hands range generally fall into two categories—purely cosmetic devices meant to replicate the look of the missing limb and functional devices that can grasp and hold objects [9]. Functional devices are further categorized by mode of operation into body-powered and myoelectric prostheses. Each type of hand has its benefits and detriments which are often contradictory across device types. Cosmetic hands are attractive and light, but cannot hold anything. Body-powered prostheses are light, durable, and provide a sense of proprioception, but require a harness that can be restricting [9]. Electric hands controlled by muscle activity in the residual limb (myoelectric hands) allow for greater arm movement and are easy to use, but seem heavy, require frequent battery changes, and don’t convey a sense of how tightly the user is gripping an object in the hand [9].

Ideally, a prosthetic hand should combine the benefits of each type of prosthesis while eliminating the detriments and be, in order of priority, light, comfortable, functional, easy to control, and run forever on a single battery. Among users of myoelectric devices, an articulated, multifunctional hand is the primary desire [3,5]. With this in mind, a review of 25 years of upper extremity prosthesis literature has shown an average 20% rejection rate across all types of devices illustrating that current hands do not meet the needs of a large portion of users [10].

Recently, more articulated hands have become commercially available including the Touch Bionic ilimb, Steeper bebionic3, and Michelangelo from Otto Bock [8,9]. These devices feature individual fingers with independent and semi-independent movement, an opposable thumb capable of circumduction, and are capable of forming a variety of grip shapes. While they are a substantial functional and cosmetic improvement over earlier myoelectric prostheses, their increased capability also results in increased weight compared to simpler myoelectric hands due their more complicated structure and increased number of motors contained in the hand.

Two highly advanced prosthetic hands currently in development include the JHU/APL Modular Prosthetic Limb (MPL) [11]developed at the Johns Hopkins University Applied Physics Lab and the “Luke” hand from DEKA [12] which recently received FDA marketing approval. Each of these device systems were developed under the Defense Advanced Research Projects Agency (DARPA) Revolutionizing Prosthetics program. Both the APL and “Luke” hands are part of their respective modular prosthetic arm systems with upwards of 22 degrees of freedom depending on the level of amputation [11,12]. These systems are able to replicate most actions of the arm and promise to afford amputees with prosthetic limbs that are more capable than current clinical options. The user interfaces for these two devices differ in that the MPL will be controlled via a neural interface still under development [13] while the DEKA arm is controlled using a foot-operated wireless orientation system in lieu of relying on EMG or shoulder motion similar to other contemporary prosthetics [6]. While most of the subjects tested with the DEKA arm enjoyed it, there was less desire to adopt such a system as the level of amputation moved distally, with weight being a chief concern [14].

Other attempts to replicate the dexterity of the human hand without regards to prosthetic applicability but as primarily robotic hands have been successful, but are far too heavy and too large to make the transition to prosthetic application. Early dexterous hands include the Salisbury Hand [15], the Utah/MIT hand [16], and DIST hand [17]. These hands used tendons such that the actuators were distant to the point of operation, keeping the hand approximately human sized. The hands proved to be dexterous (more so than a natural hand in some cases) and produced high grip forces, yet had large banks of actuators, were heavy, and had high power requirements. Other hands used intrinsic motors to directly supply joint torque and reduce the size of the actuators. This yielded either hands that were near human size, yet had weak grip strength or had high flexion force yet were larger than a normal human’s [18,19]. The Robotnaut hand developed for the International Space Station is a dexterous hand that falls in-between robotic hands and prosthetics in that it was designed to mimic a gloved human astronaut’s hand and is to be used as a teleoperated device [20]. The hand is quite capable, but still too large and heavy to be used as a prosthetic.

New dexterous and semi-dexterous hands designed primarily as prosthetic hands have been in development in recent years to address the shortcomings of currently available prosthetic hands while remaining acceptable in terms of weight, size, and appearance. Other hands under development such as the Southampton Hand [21], the MANUS-HAND [22], and that developed by Carrozza [23] use mechanical coupling linkages between the joints to replicate a natural finger arc using a single actuator. While these hands may comply to an object for an optimal grasp, many are not truly dexterous and have limited ability to conform to grasped objects. As an alternative to electric motors, fluidic actuators (using either air or water) have been explored with intriguing results [24–27]. While earlier iterations used a pump off-board the hand (located in the forearm of the prosthesis), later versions have placed a micropump within the hand itself to produce a relatively lightweight prosthesis with usable [28], if not biomimetic grip force. Other examples of experimental advanced prosthetic hands include the Cyberhand [29], IOWA hand [30], the HIT/DLR Hand [31], and the work of Massa [32] all of which use an under-actuated mechanism, with more degrees of freedom than actuators. Under-actuated hands use compliant joints and drive mechanisms, such that the individual fingers and joints can each move more independently to optimally grasp any object by conforming grasp shape to object geometry. This both reduces the number of actuators (and resulting weight and size) to one or less per finger, while affording more lifelike motion and better grip shape.

By contrast, the hand presented in this work is an over-actuated device in that it uses more actuators than it has degrees of freedom. This approach allows it to have actuators both intrinsic and extrinsic to the hand itself, more similar to a biological hand, and to be a hybrid of robotic hands with the heavier, high power actuators separate from their point of operation and small, low torque actuators directly coupled to the joints.

## Methods

The prototype prosthetic presented in this paper takes a different approach than other hands in development in that is an over-actuated device with redundant actuation for each finger. The hand operates in two distinct force spaces: a low grip force phase where grasp shape is formed, and a high grip force phase where the fingers close tightly on an object. The device is intended to capitalize on the force output of tendon driven hands while maintaining the size, flexibility, and simplicity of control of direct joint driven hands. The design is biomimetic in that it attempts to replicate most of the degrees of freedom and appearance of a human hand. The description of the device itself is broken down into three sub-sections: Physical structure, actuators, and control system.

### Structure

**Fig 1:**
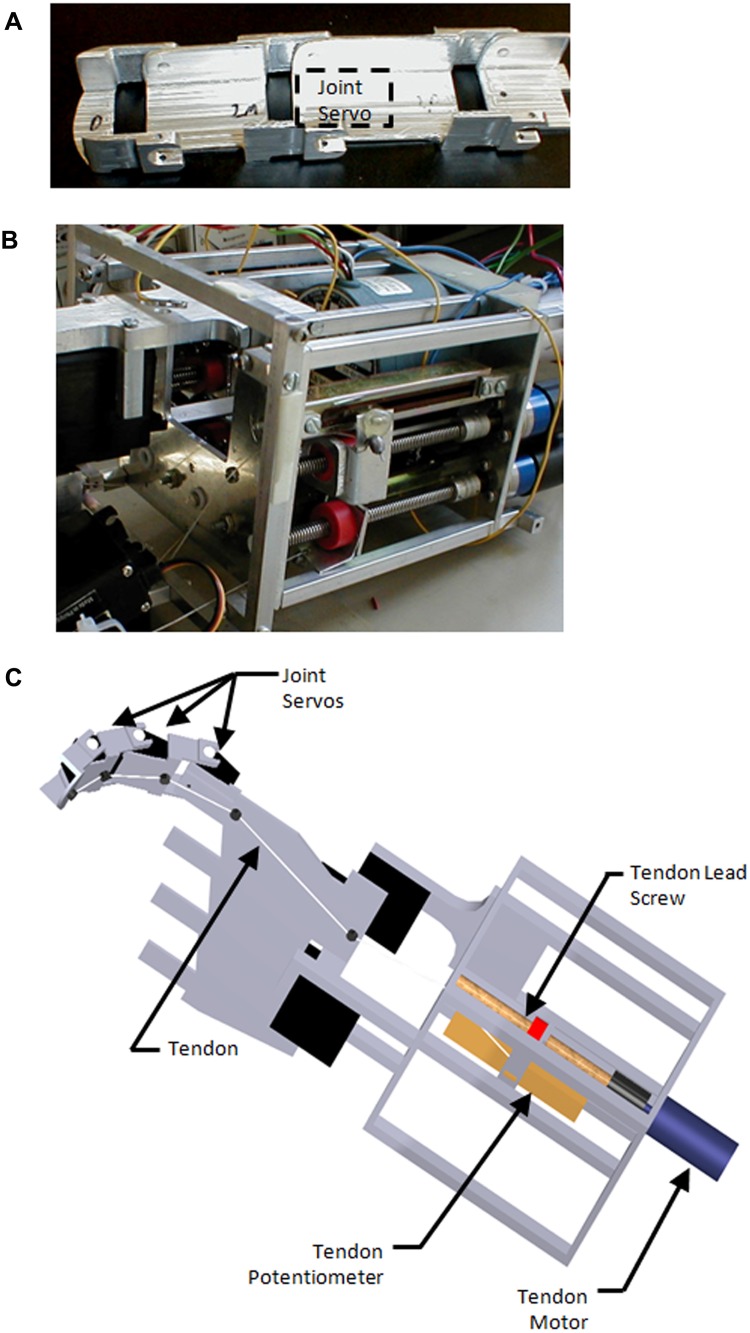
Photographs and rendering of prosthesis structure. (a) Individual finger structure with joint servo location indicated by dashed box. (b) Forearm, front oblique view illustrating front endplate and cage structure. Note tendon actuators contained within cage. The hand is attached to the left side of the forearm as seen in this picture. (c) Computer rendering of a single (index) finger and forearm illustrating the tendon routing across the palm and underside of the phalanges as well as the tendon actuation mechanism. The joint servo motors can be seen within the structure of the finger.

The structure of the hand was fabricated from aluminum to minimize weight while maintaining rigidity and strength (Fig. 1). Phalange lengths were based on a male human model, approximately 50^th^ percentile in aggregate (across all phalange measurements) as found in the work of Greiner [33]. This size was chosen for a number of reasons including convenience, adequate size for internal components, and aesthetics. The width of the fingers was determined by the size of the finger joint servo motors (21.6mm) employed in this study (Fig. 1a). The joints between phalanges were pinned on one side with the drive hub of the finger joint servo motors acting as the second pivot point of the joint (Fig. 1a,c).

The overall structure of the forearm was an open cage structure, 104mm x 88mm x 102mm in size, with solid end-plates. An open frame aluminum exoskeleton was designed such that all of the components fit within this open cage (Fig. 1b, c). This provides maximum strength with minimum size and weight. Overall length was based on the length of the actuators and the length of throw required for the finger tendons.

The palm consists of a rigid aluminum plate, approximately 1.6mm thick, which served primarily as a foundation for mounting the fingers and wrist structures. Thumb circumduction is permitted by a cut-out on the medial side of the palm (Fig. 1c). The wrist was a two degree of freedom clevis which joined the palm and forearm structures. For the purposes of this initial prototype, the wrist connecting the forearm and palm was fixed with no movement possible.

### Actuators

The novel hand used two types of actuators for each finger, a single drive tendon and individual joint servo motors. A total of 17 servo motors are used for the whole hand (the second and third fingers have no lateral degree of freedom). The servos used were Expert Electronics SL260 Sub Micro Servos, with a peak current draw of 200 mA at 5V. Able to produce 10.9 N-cm of torque and rotate 90° in 0.32 seconds, grip shape was produced quickly and accurately. Their small size (21.6mm x 11.2mm x 19.1mm) and weight (9.1g), permitted installation directly within the fingers (Fig. 1a, c).

To provide a strong grasp, a tendon system works in conjunction with the joint servos. The tendon actuators consist of small gear-head motors driving ¼-20 lead screws. This size lead screw was selected as it enables a high torque to linear force amplification without an undue loss of speed. An additional benefit of using tendons and lead screws is that the system is non-backdriveable, allowing for a strong, sustained, low power grip with the motors turned off. The motors used are MicroMo 2224–012SR coreless DC motors operating through a 3.71:1 gear reducer. Maximum torque is 7.28N-cm, drawing 1.3A at stall. The motor is able to spin at 2100 rpm with no load, moving the tendon through its entire 63.5mm throw in 1.46 seconds. Tendon throw length was determined by the amount of shortening required to travel from full extension to full flexion. The device was designed to produce a grip force of 31N at the fingertip during power grasp, approximately 1/3 of that published in the literature for maximal flexion force in able-bodied subjects [34,35], but comparable to other advanced prosthetic hands currently on the market. This choice of grip force represents a compromise between motor size and weight and functional finger force output. The tendon itself is 0.8 mm diameter aircraft cable. The routing of the tendon across the palm and under the finger phalanges can be seen in Fig. 1c.

Commercial prosthetic hands locate the actuators inside the hand or terminal device in order to be applicable to the largest range of residual limb lengths—up to those with wrist disarticulation. While more individuals can more easily potentially benefit from a self-contained hand, the location of the actuators distal from the elbow results in an unnatural weight distribution and a greater perceived “heaviness” [27] despite being lighter than a natural limb. To counter this, the use of extrinsic actuators was specifically decided upon for this prototype in order to produce a more biomimetic weight distribution and locate the bulk of the weight towards the proximal (elbow) end of the device. The use of extrinsic actuators is not novel in the design of dexterous hands [15–17,20], including some early phase prototypes of advanced prosthetic hands [11,36]. Additionally, the use of actuators extrinsic to the hand may not preclude use by individuals with longer residual limbs as covered later in the discussion.

### Control System

The prototype hand controller consists of various purchased and built distributed controllers which act in concert to produce movement and grip. Some of these controllers guide the operation of other controllers lower in the hierarchy while executing their own control functions. This distributed architecture was used to better mimic the natural system and enable possible expansion in the future (reflex control, tactile senses, etc.) of both high and low level controllers. Each finger is independent of the others, as such, the prosthetic as a whole can be viewed as a collection of autonomous cooperative robots, each with the specific task to match the control signal of the operator and grip tightly if necessary.

### Hardware

The control system for each finger uses three controllers—a master controller and two subordinates; one for the finger servos and one for controlling tendon length. The Joint Position Controller (JPC) used for each finger (and the wrist) was designed around a Basic Stamp 2SX micro-controller. The finger servo controller is a Mini SSC II and the tendon controller is a Basic Stamp 2. All subsystems are Parallax, Inc. products. Communication between subsystems is via asynchronous serial at 9600 bps.

To test the operation of the device, user input to the system comes from an instrumented control glove with potentiometers on each finger joint. While this does not reflect how the prosthetic would be used in actual application, it was necessary to provide an intuitive interface in order to properly test the capabilities of the robotic hand.

### Operation

The JPC’s uses three operational modes, an initial start-up mode to open the finger and set the tendon to the home (slack position), a calibration mode, and an active (main) mode to actually control the device. During the active mode, the device operates within two grip force spaces: zero and grasping. The logic of the JPC reflects this using two active phases: joint matching and gripping.

In the joint matching phase, the device mimics the control glove finger position with the joint servos matching the input angles. The tendon during this phase follows the amount of finger flexion/extension, proving enough slack such that the finger can move freely by the servos alone.

In the grasping phase, power to the joint servos is turned off, such that they are passive devices. Next, tendon length is decreased, pulling the fingers tight about the object. For this prototype, grip force is open-loop with the amount of tightening fixed at 90% flexion length, providing a firm grip. For prototype purposes, the controller detected an object by comparing the difference between the control glove and prototype finger positions. An object is noted as present in the prosthetic hand when the amount of flexion in the control glove is greater than the hand can achieve, due to obstruction by an object, for longer than 63ms (10 loop cycles). Upon pulling the tendon tight, the tendon motors are turned off to maintain grip force while unpowered. Once the control glove extends again after grasping, the tendon is relaxed to its previous position and the joint servos are powered back on, returning the finger to its original pre-grasping grip shape.

### Testing

In the development of a novel prosthetic hand, several key aspects of the device are of particular interest. These range from weight and performance to efficiency and aesthetics [26,27]. These aspects have been separated into the following:

1. *Size/Appearance*: Size was measured and compared to the human model. Aesthetics was interpreted subjectively based on overall appearance.
2. *Weight* of the prosthetic as a whole, as well as by components (actuators and structure) and limb segments (forearm vs. hand).
3. *Power requirements*: Current draw was measured for the hand as a whole to gauge the device’s power requirements. The current was measured under the following movement conditions.
  - Rest: The prototype is turned on, but not moving, and not being instructed to move or grip an object. The fingers are straight out in the neutral position.
  - Opening and closing: The prosthetic is actively moving from its full open position to full closed position, but not tightening about an object.
  - Full grip upon an object: The hand has gasped an object such that a tightening condition exists and is in the tendon tightening phase. The hand is pulling the tendons at maximum grip force.
4. *Grip Force*: Grip force was measured using a small load cell rigidly attached to the last phalange of the index finger. This finger was then used to pull squarely against a fixed vertical planar surface with no palm contact. Two different grips were tested: 10% flexion and 90% flexion to study the effects of grip shape on grip force. The force output of the tendon lead screw was also tested to verify tendon efficiency.
5. *Range of motion*: The hand was moved and measured through its range of motion and made to form several common shapes often performed with the hand.

## Results

### Size and Appearance

**Fig 2:**
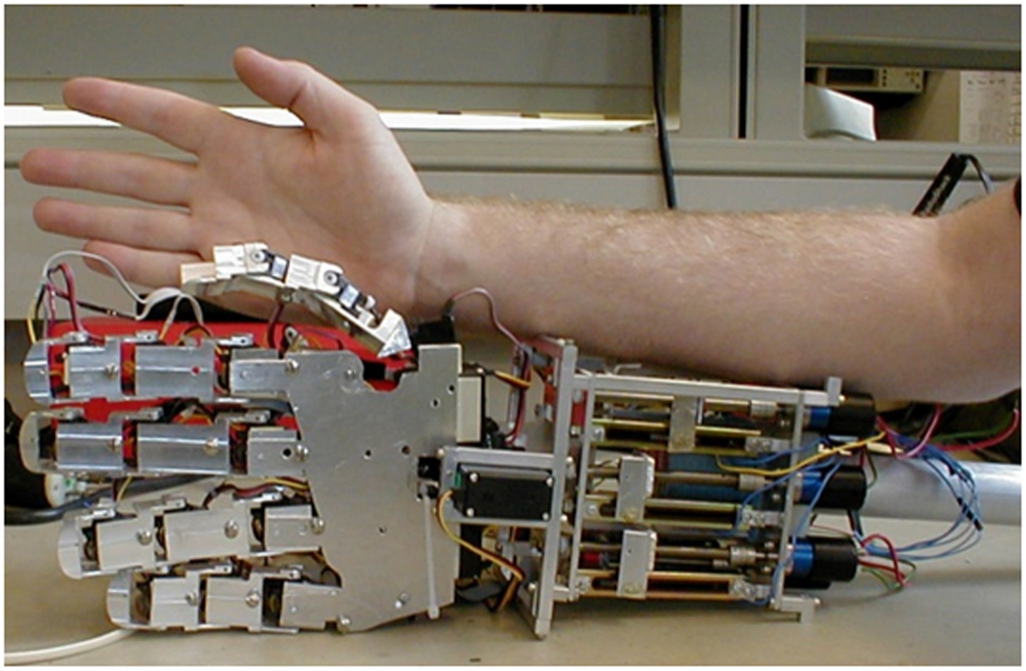
Photograph comparing the size of the over-actuated hand prototype and the human model.

The prosthetic hand was approximately 25% wider than the human model with the length matching the human model’s hand and forearm length (Fig. 2). Finger motion follows a natural finger arc while in motion, curling from the inside out.

### Weight

The overall weight of the device without controllers (which are unduly heavy for a prototype of this nature) is 1048g. Of this, the structure was about 38% (395g), and the actuators the remaining 62% (653g). In terms of limb segment, the hand with its structure and finger joint actuators was 304g (29% of total weight). The arm portion of the device weighed 744g (71% of total). The relative proportion of the hand to forearm weights was 41%. Table 1 summarizes the component and total weights. For this early phase prototype, the controllers were not optimized for weight and as such weigh approximately 500g and were not included in the overall weight analysis.

**Table 1:** Breakdown of prosthesis weight by component and limb segment.

	Component	Qty.	Unit Weight (g)	Total Weight (g)	Proportion of Total
**Structure**	Finger (avg.)	5	23	113	11%
	Palm	1	36	36	3%
	Forearm	1	245	245	23%
			Sub-Total:	395	38%
**Actuators**	Joint Servos	17	9	154	15%
	Tendon Motors & Lead Screws	5	100	499	48%
			Sub-Total:	653	62%
**Limb Segment**			Hand:	304	29%
			Arm:	744	71%
			Whole device:	1048	100%

### Power Requirements

The total resting current draw (only the controllers active) was 0.39A, with an operating draw of 3.5A for both finger motion and full powered grip. At rest, the hand required 1.8W when idle and 28W for general motion (with all finger servos in motion) and 39.2W for firmly (actively) grasping an object. Due to the dynamics of the tendon lead screws, near full grip force (90% maximum) can be maintained at the resting power of 1.8W.

### Grip Force

The maximum fingertip force observed was generated with one finger at about 90% flexion and reached a peak of 21.2N. With the finger mostly open, at 10% flexion, the fingertip force applied by the last phalange was 16.4N. The tendon drive system for each finger was able to produce 66.7N of tension during a full power grip. This is similar to the single finger grip force of 25N for the iLimb Ultra and 35N for the RSL Steeper bebionic3, though lower than average human single finger grip force of 95N [37].

### Range of Motion

**Fig 3:**
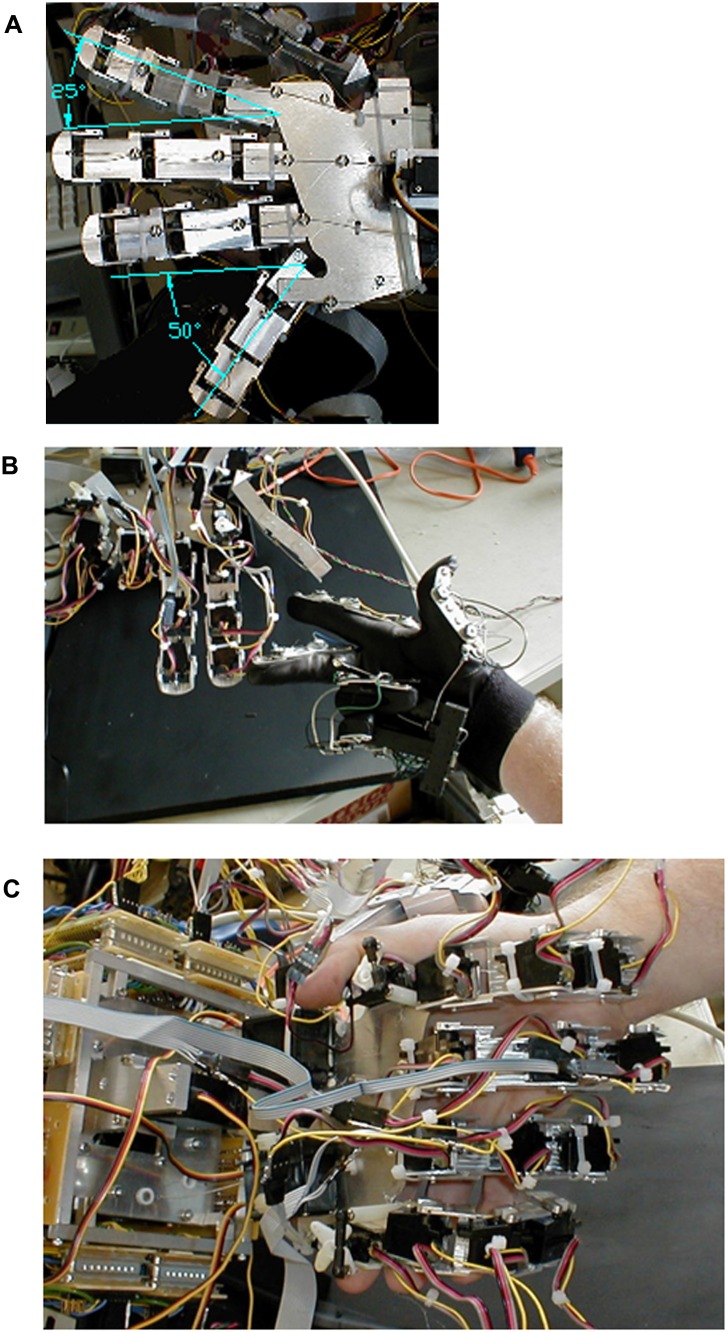
Photographs of prototype hand demonstrating range of movement. (a) Illustration of first and last finger abduction. (b) Hand mimicking user wearing control glove. (c) Hand grasping a human hand. The rear side of the fingers can be seen in this figure.

The hand was able to reproduce many common hand shapes as shown in Fig. 3. Each joint had a range from-10° (with 0° representing full natural hand extension) to about 100° at full flexion. Index and last finger abductions were 25° and 50° respectively. This range of motion is similar to natural hand range of motion of 0° (-30° for proximal joint) maximal extension, 80° 100° of joint flexion, maximum index finger abduction of 29° and last finger abduction of 45° [38]. Current semi-dexterous prosthetic hands (Ultra, bebionic3, and Michelangelo) have similar flexion/extension ranges, but do not have the ability to abduct the fingers.

## Discussion

A prototype prosthesis was developed that used a dual actuator design to allow the hand to operate in separate force modes: a low joint torque, low grip force mode with actuators at each finger joint to form grip shape, and a high grip force mode using more powerful actuators located away from the point of operation via a tendon system. This novel, over actuated system allowed for the necessarily larger, heavier components to be located more proximally for improved weight distribution and to maintain a more natural hand size and shape while producing sufficient dexterity and grip force for most activities of daily living.

### Size and Appearance

One of the main benefits of the novel design of the over-actuated hand is that it can move in more natural ways than current prosthetic hands. Finger motion follows a more graceful natural finger arc, curling the fingers at each joint, compared to both currently available devices and contemporary under-actuated artificial hands. Given the recent initiative to develop fully dexterous hands with range of motion and capability comparable to natural hands, this natural motion is critical [39]. When covered with a cover (for both appearance and protection of the device), the hand could be made to look quite acceptable to the target population. The appearance of the device could be either very natural, or very artificial, depending on the wishes of the user.

The use of commercially available joint servos results in a device where the length of the device is comparable to the human model, though the width is slightly larger than a natural hand. The choice to use “off the shelf” servos as the joint actuators was made to allow for rapid and inexpensive development of the over-actuated prototype. It is recognized that in its current state of development, the difference in width between the prototype and a natural hand would pose an issue in terms of patient acceptance as most amputees seek to maintain scale if not color or shape [40]. Refinements of the over-actuated hand would address this issue through the use of custom built finger joint servos, which could be made into the proper shape and orientation such that they fit within the dimensions of an actual finger. This task may be simplified by incorporating the gearbox housing within the phalange itself. This reduces the modularity of the prosthetic in terms of fabrication and maintenance, but would provide a way to keep the finger size as close to normal (50^th^ percentile male) as possible.

Forearm size of the prosthetic is also somewhat larger than an actual human arm. This is due mostly to the individual component nature of the linear actuators. Again, given custom built, self-contained actuators, this aspect of the prosthetic could be reduced.

### Weight

While the overall weight of the device is greater than that of clinically available prosthetics, the overall balance of the design more closely resembles that of a natural hand and arm than current clinically available prosthetic hands. Current basic myoelectric prosthetic hands weigh approximately 660g (460g for the hand itself and another 200g for socket and battery) [41], with the hand containing 70% of the total weight and being 2.3 times heavier than the forearm. This is inverted compared to natural hands which (for a 50th percentile male with a short to medium transradial amputation) have limb segment weights of 530g and 1450g for the hand and forearm respectively. Using these weights, the biological hand contains 27% of the total weight and is about a third (37%) the weight of the forearm. When more advanced dexterous hands are evaluated, the imbalance is even more striking, with the iLimb Ultra and RSLSteeper bebionic3 hands containing 72% and 75% of the hand-arm system weight respectively. Relative to the forearm, the Ultra is 2.6 times as heavy and the bebionic3 three times the weight of the forearm portion of the limb. By comparison, the hand developed in this work is more biomimetic, with most of the weight distributed closer to the rear of the device, more proximal to the elbow. This results in a more natural weight distribution with 29% of the weight in the hand, 71% in the forearm with the hand being 41% the weight of the forearm portion of the device.

This improved balance leads to a “perceived weight” that would be less than conventional options [27]. Conventional powered prosthetic hands feel “end heavy” due to the distribution of weight being more concentrated at the end of the limb in the hand. Comparing current prosthetics to the prototype hand, the maximum moment at the elbow due to the weight of a contemporary myoelectric hand is approximately 1.1Nm (again based on a 50^th^ percentile male with a short to medium length amputation), yet has a center of mass about 7mm more distal from the elbow compared to a natural arm and hand. For more dexterous prosthetics, the torque about the elbow is 1.2Nm and 1.4Nm (for the Ultra and bebionic3 respectively) with a center of mass 12mm to 18mm farther from the elbow due to their greater weight. The center of mass of the over-actuated hand is 3.8mm more proximal to the elbow, despite having a moment of 1.6Nm at the elbow. For comparison, for the same individual, the weight of a natural hand and forearm produces a maximum moment at the elbow of 3.1Nm with a center of mass about 160mm from the elbow [42]. By placing the heavier, grip force generating components in the rear of the arm/hand system, it is a better match to the feeling and balance of a natural arm. The sensation of improved balance could also be further enhanced through the use of an osseointegrated mounting post [43–45] for a more secure attachment to the residual limb though this means of fixation is still in the early stages of development. Additionally, further weight reduction could be achieved by the use of lightweight polymers or composites for potions of the structure instead of the aluminum used in the prototype to reduce the overall weight while maintaining the biomimetic weight distribution.

As the purpose of this work was primarily to assess the feasibility of the over actuated mechanism, minimizing the size and weight of the control system was not considered critical and resulted in a 500g controller. Using a more refined control system developed for later prototypes with more capable microcontrollers, an optimized circuit layout, and custom printed boards would only add an estimated 100g to the overall device (1/5^th^ the weight of the current controller) and could be located in the forearm to optimize the weight distribution.

### Power Requirements

The power requirements for the prototype are significantly considerably higher than current prosthetics (3.5A vs. 800mA) but less than that listed for similarly dexterous, commercial prosthetics (the Ultra has a maximum listed current draw of 5A) [46]. Using the battery capacities of current devices as a guide, the standard battery for the iLimb Ultra (2Ah) would allow for 34 minutes of continuous movement of the over-actuated hand, or10 minutes more than the Ultra. More basic myoelectric hands are equipped with batteries that allow for 60 minutes of movement. The DEKA arm is equipped with either an internal battery located in the forearm for an hour of operation or a larger, off-board battery (worn on the hip of the user) for 6 hours of use [12]. Given these comparable devices, the power requirements of the over-actuated hand are in-line with other prosthetic hands currently available.

Looking beyond the current prototype, refinements to the device could yield reductions in the power required to operate the hand. Combining the methods of other hands such as joint coupling [21] such that a single, small drive motor intrinsic to the hand can flex and extend each finger with a tendon drive system located in the forearm for grip force could produce reductions in not only current draw, but size, and weight as well while still providing significant grip and device balance.

### Grip Force

The tendon actuation system provided a significantly stronger grip than the intrinsic finger servos, which produced negligible grip force. The device produced an effective grip force of 21.2N per finger during power grasp, yet was less than the intended 31N individual finger grip force. This is attributed to losses within the system including off-axis routing of the tendon and friction in the tendon system. Some system losses could also be overcome by using a ball-screw in place of the lead-screw in the current prototype for more efficient power transfer. Despite being under the designed specification, the measured finger grip force is similar to the single finger grip forces of other commercially available dexterous prosthetic hands (25N for the iLimb Ultra and 35N for the RSL Steeper bebionic3) and is more than necessary to complete most tasks with a dexterous hand [28].

While grip force in the prototype hand was controlled via difference in joint position between a manual control glove and the robotic hand, in clinical operation, force feedback from finger-mounted sensors would be supplied to the user via either haptic devices [47] or via neural interface [48]. By having the user in the control loop, a more accurate grip force based on user intent can be achieved. To reduce user attention requirements, the same sensors could also be used to supply automated force control to minimize grip force while minimizing object slip [49,50].

### Range of Motion

The range of motion of the over-actuated hand is superior to both current single degree of freedom prosthetics as well as the semi-dexterous hands that are on the market. This is evident in Fig. 3, where various hand shapes are shown. The prototype at its current stage of design does posses enough flexibility to function as a replacement hand, with most grasp orientations possible. Finer motions, such as being able to touch each finger tip to the thumb (instead of just the first two) or fasten screws using just the fingers are not possible however, also being beyond the scope of other hands in development or commercially available. An additional degree of freedom in the base of the thumb is necessary for this level of motor function, an action that was left out of the prototype in order to simplify the design. By adding this ability it is expected that the prosthetic would meet the abilities of the JHU/APL, DEKA, and Robonaut hands [11,12,20] and allow for any possible hand shape to be formed.

It should be noted that the hand developed in this work, by its use of actuators extrinsic to the hand, precludes its use by very long transradial and through-wrist amputees. While this does somewhat limit its applicability, there is little benefit to having a wrist disarticulation instead of a medium to long transradial amputation [44]. It may even be more advantageous to have a transradial amputation in terms of better fit of prosthetic equipment and a larger variety of prosthesis options. While a longer residual limb is often advantageous, particularly in the lower extremities, for the arm, a more functional outcome can often be achieved with a long transradial amputation than a though wrist procedure [4]. Another option would be the use of actuators mounted to the exterior of the socket, parallel to the user’s residual limb. Such an arrangement would allow for actuators extrinsic to the hand, even for those with wrist disarticulation, while maintaining a more natural weight distribution and balance.

## Conclusion

Surveys of prosthetic hand users show that current options do not meet all of the desires of the amputee community. Previous studies indicate that users want a prosthetic hand that is attractive (natural looking while static as well as in operation), functional, comfortable, and durable. Currently available prosthetics have attempted to attend to some of these issues, yet many reasons still exist for prosthetic abandonment. The prototype dexterous hand discussed in this work addresses the need for more dexterous and capable prosthetic hands. It offers naturally curling fingers and a more human-like appearance than most currently available prosthetic options—features current users would like to see in a prosthetic hand. The use of an over-actuated mechanism allows for operation in two distinct force spaces; very fine joint torque for precise joint control while power tendons enable it to produce high flexion forces. The hand is capable of forming a variety of grasp shapes for manipulation of various shaped objects and was able to mimic the hand shape and motion of a human operator. The overall size and power requirements are comparable to other commercially available high-end prosthetic hands. Despite its slightly greater weight than conventional prosthetic hands, its center of mass is more proximal to the elbow than that of clinically available devices, and its weight distribution more closely resembles a natural arm and hand, both of which would make the device more comfortable for the user. Overall, the prototype presented in this paper demonstrates the feasibility and effectiveness of an over-actuated, biomimetic, artificial hand. It represents both a novel means of replicating the capability of the hand while remaining suitable for use as a prosthetic.
